# Automated Quality Assurance of OAR Contouring for Lung Cancer Based on Segmentation With Deep Active Learning

**DOI:** 10.3389/fonc.2020.00986

**Published:** 2020-07-03

**Authors:** Kuo Men, Huaizhi Geng, Tithi Biswas, Zhongxing Liao, Ying Xiao

**Affiliations:** ^1^University of Pennsylvania, Philadelphia, PA, United States; ^2^National Cancer Center/National Clinical Research Center for Cancer/Cancer Hospital, Chinese Academy of Medical Sciences and Peking Union Medical College, Beijing, China; ^3^UH Cleveland Medical Center, Cleveland, OH, United States; ^4^MD Anderson Cancer Center, The University of Texas, Houston, TX, United States

**Keywords:** quality assurance, OAR contouring, radiotherapy, deep active learning, clinical trial

## Abstract

**Purpose:** Ensuring high-quality data for clinical trials in radiotherapy requires the generation of contours that comply with protocol definitions. The current workflow includes a manual review of the submitted contours, which is time-consuming and subjective. In this study, we developed an automated quality assurance (QA) system for lung cancer based on a segmentation model trained with deep active learning.

**Methods:** The data included a gold atlas with 36 cases and 110 cases from the “NRG Oncology/RTOG 1308 Trial”. The first 70 cases enrolled to the RTOG 1308 formed the candidate set, and the remaining 40 cases were randomly assigned to validation and test sets (each with 20 cases). The organs-at-risk included the heart, esophagus, spinal cord, and lungs. A preliminary convolutional neural network segmentation model was trained with the gold standard atlas. To address the deficiency of the limited training data, we selected quality images from the candidate set to be added to the training set for fine-tuning of the model with deep active learning. The trained robust segmentation models were used for QA purposes. The segmentation evaluation metrics derived from the validation set, including the Dice and Hausdorff distance, were used to develop the criteria for QA decision making. The performance of the strategy was assessed using the test set.

**Results:** The QA method achieved promising contouring error detection, with the following metrics for the heart, esophagus, spinal cord, left lung, and right lung: balanced accuracy, 0.96, 0.95, 0.96, 0.97, and 0.97, respectively; sensitivity, 0.95, 0.98, 0.96, 1.0, and 1.0, respectively; specificity, 0.98, 0.92, 0.97, 0.94, and 0.94, respectively; and area under the receiving operator characteristic curve, 0.96, 0.95, 0.96, 0.97, and 0.94, respectively.

**Conclusions:** The proposed system automatically detected contour errors for QA. It could provide consistent and objective evaluations with much reduced investigator intervention in multicenter clinical trials.

## Introduction

Precise radiotherapy for optimal treatment requires the organs at risk (OARs) to be accurately delineated. Specific guidelines (1–3) that describe how OARs can be delineated reproducibly for routine clinical practice and for research purposes have been developed. Nevertheless, considerable inter- and intra-observer variations in the delineation of OARs have been reported (4–6). Such variations can result in inconsistent evaluations of the doses to the OARs, potentially hampering the efficacy of the radiotherapy and the analysis of its toxicity. Lo et al. (7) reported that 23% of contours submitted to a multicenter peer-reviewed lung stereotactic radiation therapy study needed a major revision. It is therefore essential to undertake quality assurance (QA) of OAR contouring for multicenter clinical trials because the cases come from different medical centers and so are susceptible to inconsistent contouring. This QA procedure is usually performed manually by physician investigators, which is both time-consuming and subjective. There is, therefore, a need for an automated and objective QA method.

Various approaches have been proposed for identifying contour outliners for QA purposes. Some studies (8, 9) have used statistical metrics derived from geometric distributions to determine the accuracy of the contouring; however, these metrics are not always good indicators of organ contouring. The ultimate test is to compare the contours with those that are known to be precise and accurate. The basis of such a QA method is an accurate segmentation process.

Recently, convolutional neural network (CNNs) (10, 11), a deep learning method, have proved effective for semantic segmentation, hence becoming a state-of-the-art method for this. Many studies have applied CNN to the segmentation of targets and OARs in radiotherapy for various disease sites (12–15). However, as a fully supervised deep learning method, a CNN needs a sufficiently large set of manually annotated instances to achieve good performance, and these manual annotations must be accurate enough to avoid misleading the model training. Although gold standard atlases are available (16–21), they contain few annotated cases: for example, the Lung CT Segmentation Challenge (17) includes 36 cases and the Head and Neck CT Segmentation Challenge (19) includes 48 cases. They are therefore insufficient for optimally tuning the many free parameters of the deep network. In addition, the cases submitted to the clinical trials came from different centers and contained considerable variations, and the gold standard atlases do not encompass a wide spectrum of the population. We have collected a substantial number of annotated cases in the clinical trial, with a large variation in accuracy.

In this study, we have developed a fully automated method for the QA of OAR contouring for lung cancer based on deep active learning (22–24). This uses a novel strategy that improves on the traditional CNN through an interaction between the learning algorithm and the selected noisy annotations. To the best of our knowledge, this is the first automated process for the QA of OAR contouring for multicenter clinical trials based on deep learning and the first to train a segmentation model using both active learning and large-scale noisy samples.

## Materials and Methods

### Patient Data

The patient data used in this study were a small gold standard atlas from the Lung CT Segmentation Challenge 2017 (16, 17), which included 36 cases, and 110 cases from the NRG Oncology/RTOG-1308 Trial (3). The OARs included were the heart, esophagus, spinal cord, left lung, and right lung. The manual contours of the gold standard atlas were drawn according to the RTOG-1106 contouring atlas guidelines (2), which were the same as those used for RTOG-1308 (3).

The CT scans from the Lung CT Segmentation Challenge 2017 had a reconstruction matrix of 512 × 512, with a slice thickness of 1.25–3.0 mm (median, 2.5 mm) and a pixel size of 0.98–1.37 mm (median, 0.98 mm). One of the Challenge organizers checked all the clinical contours for quality and edited them to ensure that there were no major deviations from the RTOG-1106 contouring guidelines. The CT images from RTOG-1308 were reconstructed with a matrix size of 512 × 512, a slice thickness of 1.5–3.0 mm (median, 3 mm), and a pixel size of 0.98–1.37 mm (median, 1.17 mm). The radiotherapy contours were directly drawn on the CT by radiation oncologists, but the contours from a multi-center were not all reasonable and were used for QA in this study. The data were submitted for analysis in DICOM format.

### The Automated Quality Assurance System

#### Strategy Overview

**Figure 1:**
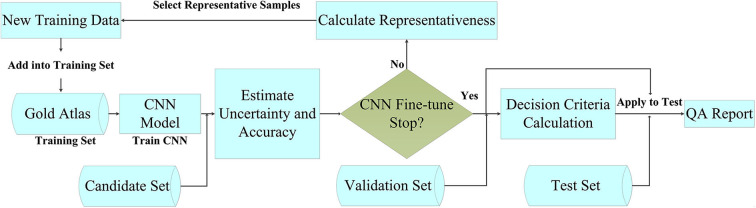
Flowchart of the contouring quality assurance strategy.

Figure 1 outlines the main concepts and the steps of our automated method for the QA of OAR delineation. The method was based on automated segmentation with deep active learning and comprised five steps: (1) We prepared a gold standard atlas for the disease site, and we divided the cases from the clinical trial into candidate, validation, and test sets. (2) We trained the CNN segmentation model with the gold standard atlas, even though this did not represent a large population. (3) To address the deficiency of the limited training data, we selected quality images from the candidate set to be added to the training set for fine-tuning of the model. The strategy for selecting these images was based on their representativeness, defined using a parameter that combined uncertainty and accuracy. (4) We evaluated the accuracy of the fine-tuned model by applying it to the validation set, which included contours with verified accuracy. The evaluation metrics included the Dice similarity coefficient (DSC) (25) and the Hausdorff distance (HD) (26). These metrics were then used to establish the QA criteria. (5) Finally, we applied the fine-tuned CNN model and the decision criteria to the test set to detect any inaccurate contours.

The QA strategy consisted of four major components: (1) a high-performance CNN for segmentation, (2) an uncertainty estimation strategy, (3) a strategy for selecting noisy annotations for fine-tuning the CNN, and (4) decision criteria. The following sections describe the active learning process in detail, focusing on the uncertainty estimation and the noisy annotation selection strategies.

##### Segmentation Model

There are many different deep neural network strategies available for segmentation. In this QA system, we used a CNN that had been demonstrated to have a state-of-the-art performance (27). The network improved the segmentation accuracy with cascaded atrous convolution and spatial pyramid pooling module. The input to the CNN was the set of original CT images, and its output was the corresponding segmentation probability maps for the OARs. To avoid overfitting, we applied general methods for data augmentation, including random scaling (from 0.5 to 1.5), random cropping, and random rotation (-5° to 5°). The optimization algorithm of training used backpropagation with stochastic gradient descent. We use the “poly” learning rate policy. We set the base learning rate to 0.001 and the power to 0.9. The batch size was set to 1 due to the limitation of physical memory on the GPU card. Every model was trained using 40 epochs. The momentum and the weight decay were set to 0.9 and 0.0005, respectively.

##### Uncertainty Estimation

A CNN model trained with only a small gold standard atlas sample will not be representative of the whole population. A straightforward strategy to improve the model was to add more images from the clinical trial. However, instead of randomly adding images to the training set, it was important to choose the most valuable images for further training. These images usually are the most confusing instances of the current model. We used an uncertainty parameter to select the images for which the CNN model had the lowest confidence so that the learning would be more effective. These images were those that lay closest to the decision boundary of the model. The uncertainty was calculated as follows:

(1) $$\begin{array}{l} U_{n\;}=\frac{1}{m\;}\overset{m}{\underset{1}{\sum }}\left(1-\max\left(p_{i},1-p_{i}\right)\right) \end{array}$$

where *U*_*n*_ denotes the uncertainty of the *n*-th image, *m* denotes the pixel number of the *n*-th image, and *p*_*i*_ denotes the probability that the *i*-th pixel belongs to the OAR to be segmented.

##### Selection of Images With Noisy Annotation

The manually drawn contours in the clinical trial showed a considerable variation, so it was not appropriate to include all of the images in the new training set. The selected images and contours should be those with higher uncertainty parameters, and the images should have accurate annotations to avoid misleading the model training. The segmentation accuracy was usually quantified using the DSC and/or the HD. The representativeness of the candidate images was assessed using a representativeness parameter (*R*_*n*_) that combined uncertainty and accuracy, which is defined as follows:

(2) $$\begin{array}{l} R_{n}=\frac{U_{n}\times DSC_{n}}{HD_{n}} \end{array}$$

where *U*_*n*_, DSC_*n*_, and HD_*n*_ denote the uncertainty, DSC, and HD of the *n*-th image, respectively. We set DSC_*n*_ = 1 and HD_*n*_ = 1 for the images without any contours (manual or predicted by the model).

After calculating the representativeness of each image, we selected the top 30% of images from the candidate set with contours and the top 30% without contours and added them to the training set for further fine-tuning. To increase the sample diversity, we set the number of iterations to two in our experiments, which added about 50% of the candidate set into the training set.

##### Decision Criteria

The accuracy of the segmentation model was evaluated on the validation set. These contours had good quality scores and were rechecked by the investigators. The slices with errors were excluded from the validation set. We used DSC and HD as the two metrics to describe the accuracy of a contour. An analysis of the contours in the validation set was performed slice by slice. Images with manual contours were used to calculate the mean and the standard deviation (σ) of both metrics for each OAR. Pass criteria were applied to all the test structures using thresholds for DSC and HD, determined as the mean either minus or plus 1.96σ, as follows:

(3) $$\begin{array}{l} DSC_{test}>mean_{DSC}-1.96\sigma_{DSC} \end{array}$$

(4) $$\begin{array}{l} HD_{test}<mean_{HD}+1.96\sigma_{HD} \end{array}$$

Small contours usually have lower DSC values but better HDs. We therefore assumed that the contours that passed either criterion were “correct.”

### Experiment and Quantitative Evaluation

This study used the 36 cases from the Lung CT Segmentation Challenge 2017 as the gold standard atlas. The 110 cases from RTOG-1308 were divided into three groups: the first 70 cases enrolled were used as the candidate set, and the remaining 40 cases were randomly assigned to validation and test sets (each with 20 cases). The gold standard atlas was used to train an initial model with reasonable performance. The candidate set was then used to enrich the training data. The accurate contours in the validation set were used to create the pass criteria. Finally, the test set was used to assess the performance of the automated QA system quantitatively using the metrics (28) balanced accuracy (BA), sensitivity, specificity, and the area under the receiving operator characteristic curve. BA quantifies a system's ability to avoid false classification and is defined as follows:

(5) $$\begin{array}{l} BA=\frac{1}{2}\left(\frac{TP}{TP+FN}+\frac{TN}{TN+FP}\right) \end{array}$$

where TP is the number of true positives, representing error-containing contours that were correctly identified, FP is the number of false positives, representing error-free contours that were mistakenly identified as having errors, TN is the number of true negatives, representing error-free contours that were correctly identified as not having errors, and FN is the number of false negatives, representing error-containing contours that were mistakenly identified as being correct.

Sensitivity evaluates the ability to identify positive labels. Specificity reflects the ability to identify negative samples. These are defined as:

(6) $$\begin{array}{l} Sensitivity=\frac{TP}{TP+FN} \end{array}$$

(7) $$\begin{array}{l} Specificity=\frac{TN}{TN+FP} \end{array}$$

## Results

### Segmentation Accuracy

The accuracy of automated segmentation was evaluated using cases in the validation set with good quality. Values of DSC and HD are shown in Table 1, with the QA pass criterion for each organ of the mean minus or plus 1.96σ. These results were at the same level with the best results of the segmentation challenge at AAPM 2017 (17). In this thoracic segmentation competition, the best DSC values were 0.93 ± 0.02 for the heart, 0.71 ± 0.12 for the esophagus, 0.89 ± 0.04 for the spinal cord, 0.98 ± 0.02 for the left lung, and 0.97 ± 0.02 for the right lung.

**Table 1:** Quantitative metrics for the organ-at-risk segmentation.

**OAR**	**DSC**		**HD (pixel)**	
	**Mean ± SD**	**Pass criterion**	**Mean ± SD**	**Pass criterion**
Heart	0.95 ± 0.03	>0.89	7.2 ± 4.1	<15.2
Esophagus	0.69 ± 0.13	>0.44	4.6 ± 2.4	<9.3
Spinal cord	0.86 ± 0.06	>0.75	2.0 ± 0.7	<3.4
Lung, left	0.96 ± 0.04	>0.88	7.3 ± 6.1	<19.3
Lung, right	0.96 ± 0.04	>0.88	7.3 ± 4.7	<16.5

*OAR, organ at risk; DSC, Dice similarity coefficient; HD, Hausdorff distance*.

### Performance for Quality Assurance

**Figure 2:**
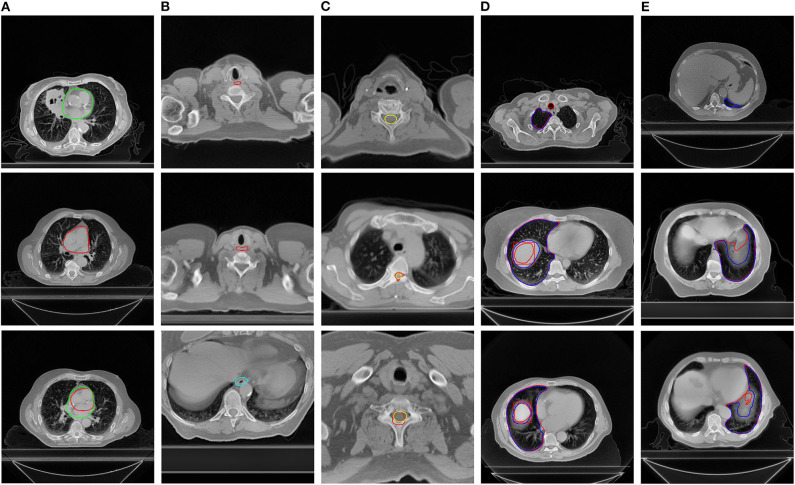
Examples of errors detected by the quality assurance system. The manual contours are in red and the automatically generated contours are in other colors. The organs at risk from the first column to the fifth column are the heart **(A)**, esophagus **(B)**, spinal cord **(C)**, right lung **(D)**, and left lung **(E)**, respectively.

Table 2 presents a list of the results of contour error detection using the test set. The BA for all the OARs was greater than 0.95, indicating that more than 95% of the contours were recognized correctly and no future manual rechecking was required. The QA for the esophagus showed lower specificity than for the other organs, but with high sensitivity. Figure 2 presents images with examples of inappropriate contours as detected by the QA system. From the first column to the fifth column are the detected errors of the heart (A), esophagus (B), spinal cord (C), left lung (D), and right lung (E), respectively. The system was able to identify slices with missing contours, contours drawn in error, and inappropriate contours.

**Table 2:** Quantitative evaluation of the contouring error detection.

**OAR**	**Number of samples**	**Number of correct samples**	**Number of incorrect samples**	**BA**	**SEN**	**SPE**	**AUC**
Heart	4,121	3,979	142	0.96	0.95	0.98	0.96
Esophagus	4,121	4,079	42	0.95	0.98	0.92	0.95
Spinal cord	4,121	3,894	227	0.96	0.96	0.97	0.96
Lung, left	4,121	4,098	23	0.97	1	0.94	0.97
Lung, right	4,121	4,094	27	0.97	1	0.94	0.97

*OAR, organ at risk; BA, balanced accuracy; SEN, sensitivity; SPE, specificity; AUC, area under the receiver operating characteristic curve*.

## Discussion

QA of OAR contouring is essential for clinical trials to reduce variability in dose evaluations and improve the outcome analysis. However, the currently used manual procedure is time-consuming and subjective, relying on the physicians' knowledge and experience, and the evaluation of the delineation is qualitative rather than based on quantitative metrics. In this study, we developed an automated contouring QA process for lung cancer based on a segmentation model trained with a deep active learning strategy. To the best of our knowledge, no similar work has previously been reported. The results of our evaluation illustrated that our QA method can efficiently differentiate correct and incorrect contours, allowing the incorrect contours to be identified automatically for further verification and revision. This could greatly improve the accuracy, efficiency, and consistency of the current process.

This new approach makes two main contributions. First, it was an active learning method that made good use of a gold standard atlas as well as information samples with uncertainties from clinical trials. Second, the CNN fine-tuning used an uncertainty estimation and a selection strategy for images with noisy annotation. The segmentation model was initially trained with the gold standard atlas. Selected high-confidence and high-uncertainty samples were then added to the training set to increase the diversity as well as the accuracy of the sample. This improved the robustness of the model, avoided biased results due to incorrect contour samples, and incorporated a larger population distribution.

Take the RTOG 1308, for example; it takes at least 5 s for a manual check of each slice, while for the proposed method it can be reduced to 0.75 s per slice (0.15 s for each OAR) with the NVIDIA Tesla K80 GPU. Compared to the conventional method, the proposed method not only saves 85% of time but also makes the evaluation more objective. For the RTOG-1308, 178 cases have been reviewed so far. The average slices of each case were about 200, so the total time saved would be about 42 h. This method is universal and can be applied to other disease sites as well. The segmentation method proposed here can be modeled using small standard samples and can also be used for automatic delineation of OARs at each center. Physicians can make minor modifications based on these automatic delineations. It is expected to improve the consistency and the efficiency of contouring for clinical trials.

There is a main limitation in this preliminary study. We did not investigate specific decision criteria for each OAR. The decision criteria were broad for some OARs and some passed contours may still need human intervention. However, the proposed method has been able to greatly improve the work efficiency compared with the manual review method. We are working on organ-specific QA criteria and hope it could further improve the evaluation accuracy.

In this study, we used the OAR contours of lung cancer to demonstrate the effectiveness of the QA strategy. Future research will include broader experiments covering all disease sites, as well as tumor targets. One potential problem may be the lack of a gold standard atlas similar to that from the Lung CT Segmentation Challenge 2017, in which case a small standard database would need to be prepared manually. Although this might be time-consuming, it would be worthwhile as great efficiency can be gained with the QA of thousands of submitted cases.

## Conclusions

The automated evaluation of contouring quality is a challenging task in radiotherapy. In this study, we described a fully automated QA system for lung cancer based on a segmentation model trained with a deep active learning strategy. This system was able to automatically detect contour errors in multicenter clinical trial data. The implementation of such a system in clinical trials could provide consistent and objective quantitative evaluations while greatly reducing investigator intervention.

## Data Availability Statement

The datasets generated and/or analyzed during the current study are not publicly available according to the Data Sharing Policy of NRG Oncology. Requests to access the datasets should be directed to NRG Oncology, APC@nrgoncology.org.

## Ethics Statement

This retrospective study was approved and carried out in accordance with the Policy of NRG Oncology. All the data are de-identified. Written informed consent for participation was not required for this study in accordance with the national legislation and the institutional requirements. The ClinicalTrials.gov Registry is NRG Oncology/RTOG 1308: NCT01993810.

## Author Contributions

All authors discussed and conceived the study design. KM wrote the programs, performed the data analysis, and drafted the manuscript. All authors discussed and made suggestions. YX guided the study and participated in the discussions and in the preparation of the manuscript. All authors read, discussed, and approved the final manuscript.

## Conflict of Interest

The authors declare that the research was conducted in the absence of any commercial or financial relationships that could be construed as a potential conflict of interest.

## Abbreviations

QA: quality assurance
OARs: organs at risk
CNN: convolutional neural networks
DSC: Dice similarity coefficient
HD: Hausdorff distance
BA: balanced accuracy
AUC: area under the receiving operator characteristic curve
TP: true positive
FP: false positive
TN: true negative
FN: false negative.

## References

[1] Kong FM Ritter T Quint DJ Senan S Gaspar LE Komaki RU. Consideration of dose limits for organs at risk of thoracic radiotherapy: atlas for lung, proximal bronchial tree, esophagus, spinal cord, ribs, and brachial plexus. Int J Rad Oncol. (2011) 81:1442–57. 10.1016/j.ijrobp.2010.07.1977 20934273 PMC3933280

[2] Kong FM Machtay M Bradley J Ten Haken R Xiao Y Matuszak M RTOG 1106/ACRIN 6697: Randomized phase II Trial of Individualized Adaptive Radiotherapy Using During Treatment FDG-PET/CT and Modern Technology in Locally Advanced Non-Small Lung Cancer (NSCLC). Philadelphia, PA: Radiation Therapy Oncology Group (2012).

[3] Liao Z Bradley J Choi N. RTOG 1308: Phase III Randomized Trial Comparing Overall Survival After Photon Versus Proton Chemotherapy for Inoperable Stage II-IIIB NSCLC. (2015). Available online at: https://www.rtog.org/ClinicalTrials/ProtocolTable/StudyDetails.aspx?study=1308

[4] Loo SW Martin WM Smith P Cherian S Roques TW. Interobserver variation in parotid gland delineation: a study of its impact on intensity-modulated radiotherapy solutions with a systematic review of the literature. Br J Radiol. (2012) 85:1070–7. 10.1259/bjr/32038456 22815411 PMC3587103

[5] Li XA Tai A Arthur DW Buchholz TA Macdonald S Marks LB. Variability of target and normal structure delineation for breast-cancer radiotherapy: a RTOG multi-institutional and multi-observer study. Int J Radiat Oncol Biol Phys. (2009) 73:944–51. 10.1016/j.ijrobp.2008.10.034 19215827 PMC2911777

[6] Nelms BE Tomé WA Robinson G Wheeler J. Variations in the contouring of organs at risk: test case from a patient with oropharyngeal cancer. Int J Radiat Oncol Biol Phys. (2012) 82:368–78. 10.1016/j.ijrobp.2010.10.019 21123004

[7] Lo AC Liu M Chan E Lund C Truong PT Loewen S. The impact of peer review of volume delineation in stereotactic body radiation therapy planning for primary lung cancer: a multicenter quality assurance study. J Thor Oncol. (2014) 9:527–33. 10.1097/JTO.0000000000000119 24736076

[8] Altman MB Kavanaugh JA Wooten HO Green OL DeWees TA Gay H. A framework for automated contour quality assurance in radiation therapy including adaptive techniques. Phys Med Biol. (2015) 60:5199. 10.1088/0031-9155/60/13/5199 26083863

[9] Chen HC Tan J Dolly S Kavanaugh J Anastasio MA Low DA. Automated contouring error detection based on supervised geometric attribute distribution models for radiation therapy: a general strategy. Med Phys. (2015) 42:1048–59. 10.1118/1.4906197 25652517

[10] Long J Shelhamer E Darrell T. Fully convolutional networks for semantic segmentation. IEEE Trans Pattern Anal Mach Intell. (2017) 39:640–51. 10.1109/TPAMI.2016.2572683 27244717

[11] He K Zhang X Ren S Sun J Deep residual learning for image recognition. Proceed IEEE Conf Comp Vis Patt Recog. (2016) 2016:770–8. 10.1109/CVPR.2016.90

[12] Ibragimov B Xing L. Segmentation of organs-at-risks in head and neck CT images using convolutional neural networks. Med Phys. (2017) 44:547–57. 10.1002/mp.12045 28205307 PMC5383420

[13] Men K Dai J Li Y. Automatic segmentation of the clinical target volume and organs at risk in the planning CT for rectal cancer using deep dilated convolutional neural networks. Medical physics. (2017) 44:6377–89. 10.1002/mp.12602 28963779

[14] Lustberg T van Soest J Gooding M Peressutti D Aljabar P van der Stoep J. Clinical evaluation of atlas and deep learning based automatic contouring for lung cancer. Radiother Oncol. (2018) 126:321–17. 10.1016/j.radonc.2017.11.012 29208513

[15] Men K Geng H Cheng C Zhong H Huang M Fan Y. More accurate and efficient segmentation of organs-at-risk in radiotherapy with convolutional neural networks cascades. Med Physics. (2019) 46:286–92. 10.1002/mp.13296 30450825 PMC6322972

[16] Yang J Sharp G Veeraraghavan H van Elmpt W Dekker A Lustberg T Data from lung CT segmentation challenge. Cancer Imag Arch. (2017). 10.7937/K9/TCIA.2017.3r3fvz08

[17] Yang J Veeraraghavan H Armato SG 3rd Farahani K Kirby JS Kalpathy-Kramer J. Autosegmentation for thoracic radiation treatment planning: a grand challenge at AAPM 2017. Med Phys. (2018) 45:4568–81. 10.1002/mp.13141 30144101 PMC6714977

[18] Clark K Vendt B Smith K Freymann J Kirby J Koppel P. The Cancer Imaging Archive (TCIA): maintaining and operating a public information repository. J Digit Imag. (2013) 26:1045–57. 10.1007/s10278-013-9622-7 23884657 PMC3824915

[19] Raudaschl PF Zaffino P Sharp GC Spadea MF Chen A Dawant BM. Evaluation of segmentation methods on head and neck CT: auto-segmentation challenge 2015. Med Phys. (2017) 44:2020–36. 10.1002/mp.12197 28273355

[20] Pekar V Allaire S Qazi AA Kim JJ Jaffray DA Head and neck auto-segmentation challenge: segmentation of the parotid glands. In: Medical Image Computing and Computer Assisted Intervention (MICCAI). Beijing; Berlin; Heidelberg: Springer (2010). p. 273–80

[21] Nyholm T Svensson S Andersson S Jonsson J Sohlin M Gustafsson C. MR and CT data with multiobserver delineations of organs in the pelvic area-Part of the Golden atlas project. Med Phys. (2018) 45:1295–300. 10.1002/mp.12748 29322528

[22] Prince M Does active learning work? A review of the research. J Engin Educ. (2004) 93:223–31. 10.1002/j.2168-9830.2004.tb00809.x

[23] Dutt Jain S Grauman K Active image segmentation propagation. In: Proceedings of the IEEE Conference on Computer Vision and Pattern Recognition. Las Vegas, NV: IEEE (2016). p. 2864–73. 10.1109/CVPR.2016.313

[24] Yang L Zhang Y Chen J Zhang S Chen DZ Suggestive annotation: a deep active learning framework for biomedical image segmentation. In: International Conference on Medical Image Computing and Computer-Assisted Intervention. Cham: Springer (2017). p. 399–407. 10.1007/978-3-319-66179-7_46

[25] Crum WR Camara O Hill DLG. Generalized overlap measures for evaluation and validation in medical image analysis. IEEE Trans Med Imag. (2006) 25:1451–61. 10.1109/TMI.2006.880587 17117774

[26] Huttenlocher D P Klanderman GA Rucklidge WJ Comparing images using the Hausdorff distance. IEEE Trans Pattern Anal Mach Intell. (1993) 15:850–63. 10.1109/34.232073

[27] Men K Boimel P Janopaul-Naylor J Zhong H Huang M Geng H. Cascaded atrous convolution and spatial pyramid pooling for more accurate tumor target segmentation for rectal cancer radiotherapy. Phys Med Biol. (2018) 63:185016. 10.1088/1361-6560/aada6c 30109986 PMC6207191

[28] Sokolova M Lapalme G A systematic analysis of performance measures for classification tasks. Inform Proc Manag. (2009) 45:427–37. 10.1016/j.ipm.2009.03.002

